# Multiplex complement profiling uncovers complement activation signatures in microvascular manifestations of antiphospholipid syndrome

**DOI:** 10.1016/j.rpth.2026.106996

**Published:** 2026-10-01

**Authors:** Bin Cai, Can Huang, Yulan Chen, Yangzhong Zhou, Yuan Zhao, Xiaoyue Deng, Mengtao Li, Xiaofeng Zeng, Jiuliang Zhao

**Affiliations:** 1 Department of Rheumatology and Clinical Immunology, Peking Union Medical College Hospital, Chinese Academy of Medical Sciences, Peking Union Medical College, Beijing, China; 2 National Clinical Research Center for Dermatologic and Immunologic Diseases (NCRC-DID), Ministry of Science & Technology, Beijing, China; 3 State Key Laboratory of Complex Severe and Rare Diseases, Peking Union Medical College Hospital, Beijing, China; 4 Key Laboratory of Rheumatology and Clinical Immunology, Ministry of Education, Beijing, China; 5 Department of Nephrology, Zhongshan Hospital, Fudan University, Shanghai, China; 6 Department of Rheumatology and Immunology, Shenzhen People’s Hospital (The First Affiliated Hospital, Southern University of Science and Technology; The Second Clinical Medical College, Jinan University), Shenzhen, Guangdong, China

**Keywords:** complement activation, microvascular antiphospholipid syndrome, multiplex complement profiling, cluster analysis, predictive model

## Abstract

**Background:**

The 2023 American College of Rheumatology/European League Against Rheumatism antiphospholipid syndrome (APS) classification criteria define a clinical domain characterized mainly by microvascular thrombosis formation and is associated with complement activation.

**Objective:**

We aimed to evaluate the relationship between the complement profile and organ-specific manifestations of APS, especially microvascular APS (MAPS).

**Methods:**

Patients who fulfilled the 2023 ACR/EULAR APS classification criteria were enrolled. Plasma levels of complement components (Ba, Bb, C3a, C4a, C5a, soluble C5b-9, factor H, factor I, C1q, C2, C3, C4, C5, factor D, and factor P) were quantified by chemiluminescent multiplex enzyme-linked immunosorbent assays. Cluster analysis was performed to identify distinct complement activation profiles among clinical subgroups.

**Results:**

A total of 58 patients with APS, including 51 patients with MAPS consecutively enrolled between August 2023 and June 2025, and 20 healthy controls (HCs) were included in the study. Compared with HCs, patients with MAPS had significantly higher plasma levels of Ba, C3a, C4a, and sC5b-9, and significantly lower levels of C1q, C2, and factor P (all *P* < .05). Cluster analysis identified 3 subgroups: cluster 1 characterized by predominant MAPS manifestations with marked alternative pathway activation; cluster 2 enriched for myocardial disease and antinuclear antibody positivity, showing predominance of classical and lectin pathway activation; and cluster 3 characterized by macrovascular thrombosis with relatively mild complement activation. A predictive model combining factor P and C3a demonstrated good performance for MAPS (AUC = 0.898; sensitivity 82.4%; specificity 88.9%).

**Conclusions:**

Complement profiling may serve as a valuable tool for early identification of MAPS, disease monitoring, and guiding individualized treatment, highlighting the potential of complement-targeted therapies in MAPS.

## Essentials

• Microvascular APS manifestations are recognized in the 2023 ACR/EULAR criteria.
• Complement profiling and clustering revealed distinct patterns across APS phenotypes.
• MAPS showed increased Ba, C3a, C4a, and sC5b-9 but decreased C1q, C2, and factor P.
• Factor P plus C3a identified MAPS accurately (AUC 0.898).

## Graphical abstract

**Figure:**
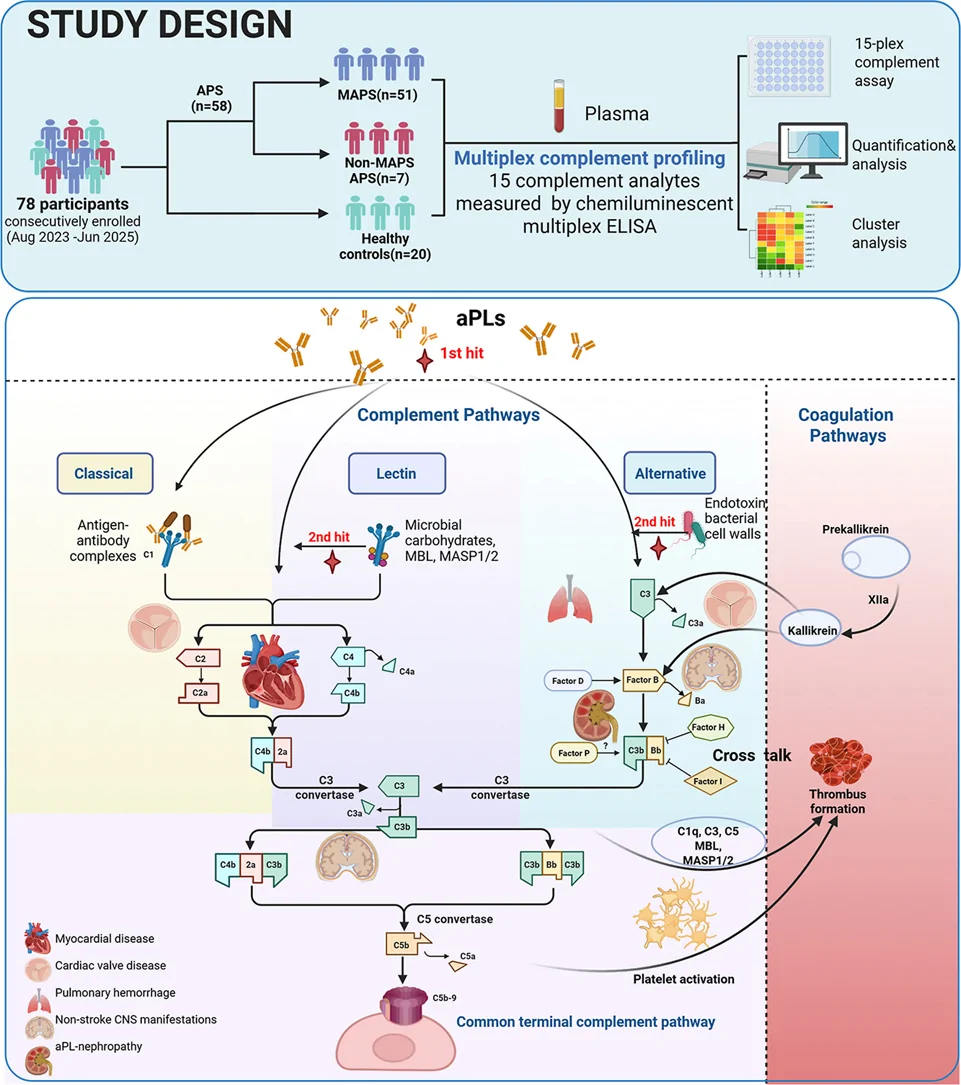

**Key messages:**

**What is already known on this topic**

Complement dysregulation activation is one of the important pathogenic mechanisms of antiphospholipid syndrome, especially in microvascular antiphospholipid syndrome. However, these studies lack multi-complement profiling, and thus cannot characterize the specific activated complement pathway.

**What this study adds**

1. Complement activation across the classical, lectin, and alternative pathways is closely associated with MAPS, and especially the alternative pathway playing a particularly prominent role.
2. Distinct complement activation patterns were observed across different MAPS-related organ involvements.
3. Through cluster analysis, the complement level can effectively classify APS into 3 clinical subtypes.
4. A predictive model combining factor P and C3a demonstrated good performance for MAPS (AUC = 0.898; sensitivity 82.4%; specificity 88.9%).

**How this study might affect research, practice or policy**

1. This study may inform risk stratification for MAPS and ultimately facilitate the integration of complement-targeted therapies into routine clinical practice.
2. This research will incite further investigation into complement’s connection with MAPS and other rheumatic immune diseases.

## 1 Introduction

Antiphospholipid syndrome (APS) is an autoimmune disease with a complicated antiphospholipid antibodies (aPLs)-mediated pathogenesis, which is characterized by venous and arterial thrombosis, pregnancy morbidity, and a wide spectrum of microvascular and nonthrombotic manifestations [1]. Despite advances in management, APS continues to carry substantial mortality, particularly in the form of catastrophic APS (CAPS), which is characterized by widespread microvascular involvement affecting multiple organ systems and is associated with a reported mortality rate of ∼56% during a 10-year follow-up period [2].

Microvascular manifestations of APS (MAPS) refers to a spectrum of clinical manifestations characterized by progressive intimal proliferation leading to occlusion of small vessels across multiple organs, largely driven by abnormal proliferation of endothelial and smooth muscle cells in the presence of aPLs [3], [4], [5]. MAPS has been newly incorporated into the updated 2023 APS classification criteria and represents a clinically and mechanistically distinct phenotype from moderate-to-large vessel diseases [6]. It encompasses manifestations such as livedoid vasculopathy, aPL nephropathy, pulmonary hemorrhage, myocardial disease, and adrenal hemorrhage or microthrombosis. MAPS is estimated to occur in up to 12% of patients with persistently positive aPLs worldwide [7]. Patients with MAPS not only have a poorer prognosis but also have an increased risk of relapse and a greater need for additional therapeutic interventions [8]. Apart from the adverse prognosis caused by major organ damage, another major determinant of the poor prognosis in MAPS is diagnostic delay. Current classification criteria require histopathological confirmation in addition to imaging evidence, yet invasive procedures involving vital organs are often restricted in these patients due to ongoing anticoagulant or antiplatelet therapy. Therefore, exploring noninvasive biomarkers, such as peripheral blood-derived markers, is crucial for the timely recognition of MAPS.

The complement system has long been recognized as a key effector of innate immunity, mediating host defense and tissue homeostasis. It can be triggered through classical, alternative, and lectin pathways, all converging on C3 and ultimately leading to the generation of C5a and the membrane attack complex (C5b–9). Dysregulated complement activation contributes to the development of multiple autoimmune diseases [9], and plasma complement profiles may therefore serve as indicators of disease activity [10]. Emerging evidence underscores the pivotal role of complement activation in the pathogenesis of APS [11], particularly MAPS. Clinical and pathologic studies have shown that patients who are at a high risk for CAPS, even during remission, exhibit persistently elevated circulating C5a and soluble C5b-9 (sC5b-9) levels, and detectable C5b-9 deposition in the affected microvascular endothelium [12], [13], [14], [15], suggesting a more severe disease phenotype and a higher risk of relapse. Moreover, real-world evidence showing clinical responses to terminal complement blockade with eculizumab in refractory or relapsing APS provides additional treatment-based support for considering complement activation products as candidate biomarkers for therapeutic prediction and disease monitoring [16].

Complement activation has been neglected in the pathogenesis of APS. Although current evidence generally supports its involvement in MAPS, most studies have been limited to the detection of terminal complement components such as C5 and C5b–9, which cannot reflect the activation status of the 3 complement pathways in patients with MAPS. This study aimed to comprehensively assess the relationship between complement activation and the clinical phenotypes of APS, particularly MAPS, by measuring 15 complement components, and to establish a predictive model for MAPS based on complement biomarkers. Our findings may inform risk stratification for MAPS and ultimately facilitate the integration of complement-targeted therapies into routine clinical practice.

## 2 Methods

### 2.1 Study population

This study was based on a prospective APS cohort at Peking Union Medical College Hospital (PUMCH). Between August 2023 and June 2025, 58 patients fulfilling the 2023 American College of Rheumatology/European League Against Rheumatism (ACR/EULAR) classification criteria for APS and 20 age- and sex-matched healthy controls (HCs) were enrolled. Sex was defined as biological sex (male or female) based on information recorded in the patients' medical records. Individuals with active infection at the time of blood sampling were excluded. Among the patients with APS, 51 were classified as MAPS based on the presence of at least one microvascular manifestation, regardless of concomitant macrovascular thrombosis, whereas the remaining 7 patients were classified as non-MAPS. MAPS manifestations included livedoid vasculopathy, aPL nephropathy, pulmonary hemorrhage, myocardial disease, as well as retinal microvascular manifestations and nonstroke central nervous system (CNS) manifestations [17], [18], [19], [20].

Baseline demographic, clinical, laboratory, and treatment data were systematically collected. Lupus anticoagulant (LA), anticardiolipin (aCL), and anti-β2-glycoprotein I (aβ2GPI) antibodies were assessed as previously described [17]. aCL and anti-β2GPI antibodies were measured using enzyme-linked immunosorbent assay (ELISA) (QUANTA Lite, INOVA Diagnostics). Moderate antibody titers were defined as values ranging from 40 to 79 units, whereas high titers were defined as values ≥80 units. Autoantibody profiles, including anti–double-stranded DNA and anti–extractable nuclear antigen antibodies, were reviewed, and patients were assessed for coexisting connective tissue diseases, including systemic lupus erythematosus (SLE), according to established classification criteria. This study was approved by the Medical Ethics Committee of PUMCH (HS-3309), and written informed consent was obtained from all participants.

### 2.2 Sample collection

Peripheral blood samples were collected in EDTA-containing tubes at the time of study enrollment for complement analysis. Samples were centrifuged at 1000 g for 10 minutes, and plasma was transferred to a new tube and centrifuged again at 12,000 g for 2 minutes. Following processing, plasma samples were aliquoted and immediately stored at −80°C [21].

### 2.3 Complement measurement

To quantify complement levels (Ba, Bb, C3a, C4a, C5a, sC5b-9, factor H, factor I, C1q, C2, C3, C4, C5, factor D, and factor P), plasma samples were thawed at 37°C (water bath) and immediately transferred to ice to prevent complement activation before processing. Plasma complement was detected using a chemiluminescent multiplex ELISA per manufacturer’s recommendations (Quidel; cat. numbers: A900, A916). Images were captured using a Q-View imager to calculate complement levels. Manufacturer-provided control samples were included on each plate to ensure plate-to-plate consistency [22].

### 2.4 Cluster analysis

Before cluster analysis, continuous variables were standardized. For parameters typically reduced in disease (C1q, C2, C3, C4, C5, factor D, and factor P), values were negated before clustering to facilitate interpretation. Hierarchical clustering was performed using Euclidean distance and Ward linkage. The ‘NbClust’ package in R software was used to determine the optimal number of clusters based on multiple clustering indices [23], [24]. Data visualization was performed using the ‘pheatmap’ and ‘ggpubr’ packages.

### 2.5 Statistical analysis

For continuous variables, data are described as mean ± SD or median and IQR. Comparisons among multiple groups were performed using anova or the Kruskal–Wallis H test, and between 2 groups with Student’s *t*-test or the Mann–Whitney U-test. For categorical variables, data are presented as counts and frequencies, and differences were assessed using the chi-squared test.

Associations between plasma complement levels and MAPS were examined using logistic regression analyses. Model 1 was unadjusted, Model 2 was adjusted for age and sex, and Model 3 was further adjusted for anticoagulant therapy, antiplatelet therapy, glucocorticoid use, immunosuppressive treatment, and hydroxychloroquine exposure.

Complement components with significant differences among MAPS patients (C1q, C2, Ba, C3a, C4a, factor P, and sC5b-9) were entered into a multivariate logistic regression model to identify the optimal combination of predictors. The discriminant ability of the predictive model was evaluated using the receiver operating characteristic (ROC) curve analysis. Model accuracy was further verified by bootstrap validation using computer resampling for 1000 repetitions of simple random sampling with replacement; model consistency was evaluated by generating calibration curves (Supplementary Figure 1).

All statistical analyses were performed using R software (version 4.2.0), SPSS (version 22.0; IBM), and Empower Stats software (www.empowerstats.com, X&Y Solutions). A 2-sided *P* < 0.05 was considered statistically significant.

## 3 Results

### 3.1 Baseline characteristics

As demonstrated in Table 1, a total of 58 patients with APS were included in the study, of whom 37 (63.8%) were female, with a median age of 37.0 years. Twenty-one patients (36.21%) had positive antinuclear antibody (ANA) and fulfilled the classification criteria for SLE. There was no significant difference in sex or age between patients with APS and HCs (*P* > 0.05).

**Table 1:** Baseline clinical characteristics of the study population

	APS			HC	*P* value
	Total	MAPS	Non-MAPS APS		
N (%)	58	51	7	20	
Female, n (%)	37 (63.79)	34 (66.67)	3 (42.86)	13 (65.00)	.47
Age (years) (median (IQR)	37.00 (30.50-43.75)	37.00 (30.00-43.00)	33.00 (32.50-50.50)	28.50 (25.00-41.75)	.27
ANA positive, n (%)	21 (36.21)	20 (39.22)	1 (14.29)	N/A	.40
Venous thrombosis, n (%)	26 (44.83)	21 (41.18)	5 (71.43)	N/A	.23
Arterial thrombosis, n (%)	26 (44.83)	22 (43.14)	4 (57.14)	N/A	.69
Pregnancy morbidity§, n (%)	4 (10.81)	4 (11.76)	0 (0.00)	N/A	.44
Cardiac valve disease, n (%)	19 (32.76)	18 (35.29)	1 (14.29)	N/A	.41
Thrombocytopenia, n (%)	27 (46.55)	24 (47.06)	3 (42.86)	N/A	.83
Microvascular disease, n (%)					
Livedo racemosa	19 (32.76)	19 (37.25)	N/A	N/A	N/A
aPL nephropathy	11 (18.97)	11 (21.57)	N/A	N/A	N/A
Pulmonary hemorrhage	4 (6.90)	4 (7.84)	N/A	N/A	N/A
Myocardial disease	13 (22.41)	13 (25.49)	N/A	N/A	N/A
Retinal microvascular manifestations	4 (6.90)	4 (7.84)	N/A	N/A	N/A
Nonstroke CNS manifestations	15 (25.86)	15 (29.41)	N/A	N/A	N/A
APL profiles, n (%)				N/A	
Positive LA (persistent)	48 (82.76%)	41 (80.39)	7 (100.00)	N/A	.34
aCL IgM medium/high titer	4 (6.90%)	2 (3.92)	2 (28.57)	N/A	.07
aCL IgG medium/high titer	24 (41.38%)	22 (43.14)	2 (28.57)	N/A	.69
aβ2GPI IgM medium/high titer	11 (18.97%)	9 (17.65)	2 (28.57)	N/A	.61
aβ2GPI IgG medium/high titer	29 (50.00%)	27 (52.94)	2 (28.57)	N/A	.42
Treatments, n (%)				N/A	
Antiplatelet	24 (41.38%)	21 (41.18)	3 (42.86)	N/A	.93
Anticoagulant	37 (63.79%)	32 (62.75)	5 (71.43)	N/A	.65
Hydroxychloroquine	46 (79.31%)	39 (76.47)	7 (100.00)	N/A	.33
Glucocorticoid	39 (67.24%)	36 (70.59)	3 (42.86)	N/A	.25
Immunosuppressant	30 (51.72%)	28 (54.90)	2 (28.57)	N/A	.20
Complement test (median, IQR)					
C1q (μg/mL)	85.65 (77.16–101.86)	84.86 (76.38–101.05)	91.14 (78.95–111.34)	95.88 (87.42–109.95)	.08
Intact C2 (μg/mL)	15.83 (12.46–20.54)	14.72 (12.30–19.19)	20.65 (18.07–28.98)	21.06 (19.12–25.52)	<.01
Intact C3 (μg/mL)	844.70 (807.40–926.71)	840.71 (804.07–916.23)	863.62 (842.36–979.57)	863.29 (826.86–929.24)	.49
Intact C4 (μg/mL)	292.63 (290.46–295.48)	292.42 (290.48–295.45)	292.84 (291.11–298.42)	294.99 (291.92–297.06)	.24
Intact C5 (μg/mL)	129.57 (112.42–152.42)	128.57 (112.96–152.28)	140.94 (114.55–156.57)	121.41 (112.76–151.55)	.91
Factor D (μg/mL)	0.85 (0.65–1.19)	0.84 (0.66–1.17)	0.95 (0.65–1.14)	0.76 (0.61–0.87)	.36
Factor P (μg/mL)	22.21 (18.29–29.08)	21.73 (17.82–28.65)	25.46 (24.88–35.11)	34.54 (27.95–38.26)	<.01
Ba (ng/mL)	586.57 (417.52–751.69)	593.14 (464.42–765.04)	449.94 (368.46–558.39)	442.87 (331.07–499.22)	<.01
Bb (μg/mL)	1.27 (0.88–1.70)	1.29 (0.90–1.74)	1.04 (0.82–1.44)	1.06 (0.80–1.28)	.22
C3a (ng/mL)	53.62 (38.06–75.68)	56.84 (40.43–79.13)	37.80 (28.93–41.83)	28.77 (23.88–31.74)	<.01
C4a (ng/mL)	295.44 (205.98–455.79)	300.06 (211.47–452.75)	211.93 (176.72–451.09)	197.48 (156.48–237.47)	<.01
Factor I (μg/mL)	25.42 (19.37–28.15)	25.45 (20.61–27.84)	17.81 (14.44–28.35)	23.06 (19.09–27.24)	.47
Factor H (μg/mL)	278.44 (253.03–325.50)	278.73 (249.56–323.53)	278.14 (264.33–321.29)	277.66 (239.36–312.21)	.79
C5a (ng/mL)	7.25 (5.11–9.15)	7.25 (5.50–9.63)	4.33 (3.32–8.10)	8.32 (7.09–9.73)	.24
sC5b-9 (ng/mL)	218.05 (176.00–373.24)	238.60 (176.00–385.32)	176.70 (176.00–247.77)	177.08 (176.00–226.04)	.09

APS, antiphospholipid syndrome; MAPS, microvascular antiphospholipid syndrome; HC, healthy control; ANA, antinuclear antibody; aPL, antiphospholipid antibody; aCL: anticardiolipin antibody; aβ2GPI, anti-β2-glycoprotein I; LA, lupus anticoagulant; CNS, central nervous system; IQR, interquartile range; N/A, not available or not applicable.

§ Meeting the pregnancy morbidity criteria of the 2006 revised Sydney APS classification.

Patients with APS were divided into MAPS (*n* = 51) and non-MAPS APS (*n* = 7) groups, with no significant difference in sex or age. Six clinical domains were clustered in the 2023 ACR/EULAR classification criteria for APS [6]. Although concomitant macrovascular thrombosis remained common in the MAPS group, patients with MAPS had lower frequencies of venous (71.43% vs 41.18%) and arterial thrombosis (57.14% vs 43.14%) but higher frequencies of pregnancy morbidity (0% vs 11.76%) and cardiac valve diseases (14.29% vs 35.29%) compared with patients with non-MAPS APS. In addition, in patients with MAPS, the most common manifestations were livedo racemose (37.25%) and nonstroke CNS manifestations (29.41%), followed by myocardial disease (25.49%) and aPL-nephropathy (21.57%).

Although the differences were not statistically significant, patients with MAPS were less frequently positive for medium-to-high titers of aCL IgM (3.92% vs 28.57%) and aβ2GPI IgM (17.65% vs 28.57%) but more frequently positive for aCL IgG (43.14% vs 28.57%) and aβ2GPI IgG (52.94% vs 28.57%). In terms of treatments, glucocorticoids (70.59% vs 42.86%) and immunosuppressants (54.90% vs 28.57%) were more frequently used in patients with MAPS, whereas anticoagulants and hydroxychloroquine were more frequently used in the non-MAPS APS group.

### 3.2 Comparison of plasma complement components across subgroups

Absolute complement concentrations are shown in Table 1, whereas standardized values were used for subgroup comparison in Figure 1. As shown in Figure 1A, patients with MAPS showed a distinct complement activation pattern compared with HCs, with higher levels of Ba, C3a, C4a, and sC5b-9, along with lower levels of C1q, intact C2, and factor P (all *P* < .05). Compared with patients with non-MAPS APS, those with MAPS exhibited significantly decreased levels of intact C2 and elevated levels of C3a (both *P* < .05); lower factor P levels and higher Ba and Bb levels were also found in the MAPS group, although the differences did not reach statistical significance.

**Figure 1:**
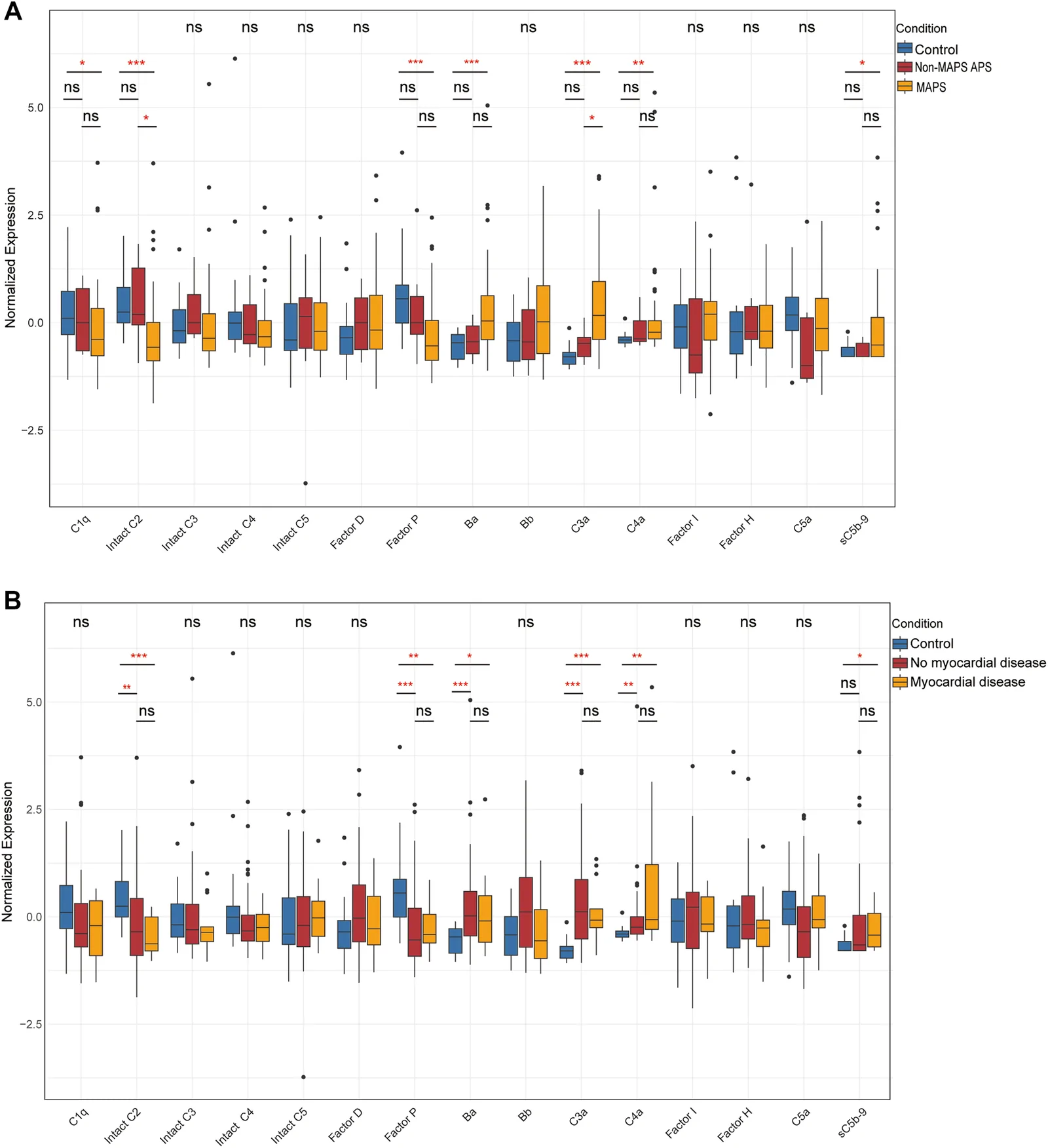
Comparison of plasma complement levels according to MAPS status and myocardial disease. (A) Complement levels of patients with MAPS, non-MAPS antiphospholipid syndrome, and HCs; (B) complement levels of patients with or without myocardial disease and HCs. ∗*P* < .05; ∗∗ *P* < .01; ∗∗∗ *P* < .001. HC, healthy control; MAPS, microvascular antiphospholipid syndrome; NS, no significance.

We constructed 3 multivariable regression models to investigate the independent role of plasma complement levels in the occurrence of MAPS. In Model 1, no covariates were adjusted. Model 2 was adjusted for age and sex. Model 3 was further adjusted for anticoagulant therapy, antiplatelet therapy, glucocorticoid use, immunosuppressive treatment, and hydroxychloroquine exposure. In both Models 1 and 2, complement C2, factor P, Ba, Bb, C3a, C4a, and sC5b-9 were significantly associated with MAPS. After further adjustment for multiple baseline covariates in Model 3, complement C2, factor P, Ba, and C3a remained significantly associated with MAPS (Supplementary Table 1).

Subgroup analyses were performed according to specific organ involvement. Compared with HCs, patients with or without myocardial disease had significantly lower levels of intact C2 and factor P, together with higher levels of Ba, C3a, and C4a. Increased sC5b-9 levels were observed only in patients with myocardial disease (all *P* < .05; Figure 1B).

In the nonstroke CNS analysis, both APS subgroups had lower intact C2 and factor P levels and higher Ba, C3a, and C4a levels compared with HCs. Elevated Bb was specific to patients with nonstroke CNS manifestations, whereas lower C1q levels were only observed in patients without nonstroke CNS manifestations (all *P* < .05). Furthermore, factor D was statistically elevated in the patients with nonstroke CNS manifestations compared with both HCs and individuals without nonstroke CNS manifestations (both *P* < .05, Figure 2A).

**Figure 2:**
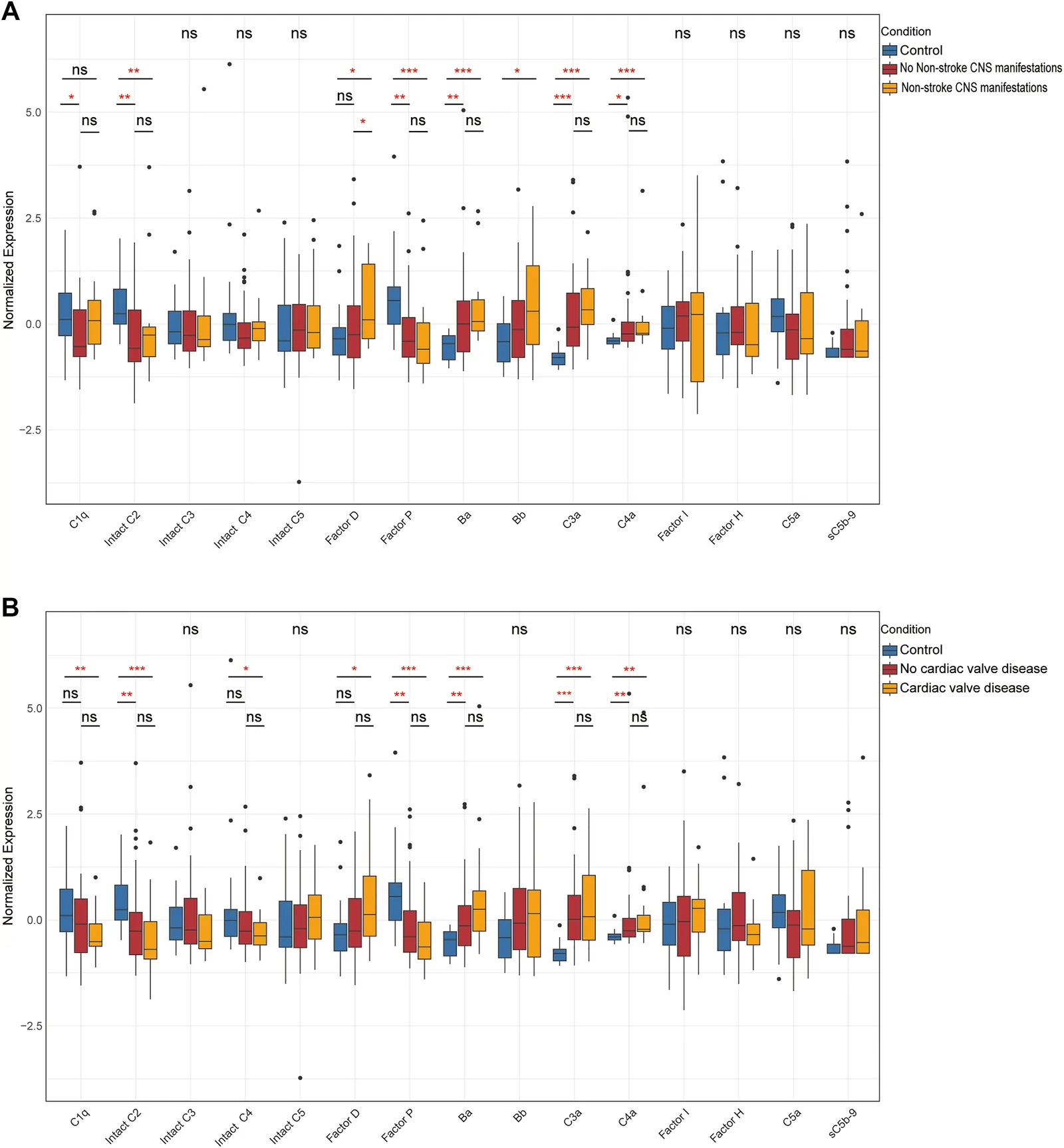
Comparison of plasma complement levels according to non-stroke CNS manifestations and cardiac valve disease. (A) Complement levels of patients with or without non-stroke CNS manifestations and healthy controls; (B) complement levels of patients with or without cardiac valve disease and healthy controls. ∗*P* < .05; ∗∗ *P* < .01; ∗∗∗ *P* < .001. CNS, central nervous system. NS, no significance.

Cardiac valve disease has been one of the ‘non-criteria’ manifestations, being associated with MAPS. Compared with HCs, patients with or without cardiac valve disease had significantly lower levels of intact C2 and factor P, together with higher Ba, C3a, and C4a levels. Patients with cardiac valve disease also had statistically decreased C1q and intact C4, but increased factor D (all *P* < .05, Figure 2B).

### 3.3 Associations between complement components and MAPS-related manifestations

A Sankey diagram was further utilized to elucidate the strength of associations between complement components and MAPS-related manifestations (Figure 3). Overall, complement alterations across the classical, alternative, and terminal pathways were associated with distinct MAPS-related manifestations. Intact C2, C4a, factor P, and Ba showed associations with multiple MAPS manifestations. Lower C1q levels were associated with MAPS and cardiac valve disease, whereas lower intact C4 levels were mainly associated with cardiac valve disease and aPL nephropathy. Increased factor D levels were associated with nonstroke CNS manifestations, cardiac valve disease, and aPL nephropathy, whereas elevated Bb levels were mainly associated with aPL nephropathy. Increased sC5b-9 levels were observed predominantly in patients with MAPS and myocardial disease. These results suggest that all 3 complement pathways contribute to the pathogenesis of MAPS-related manifestations.

**Figure 3:**
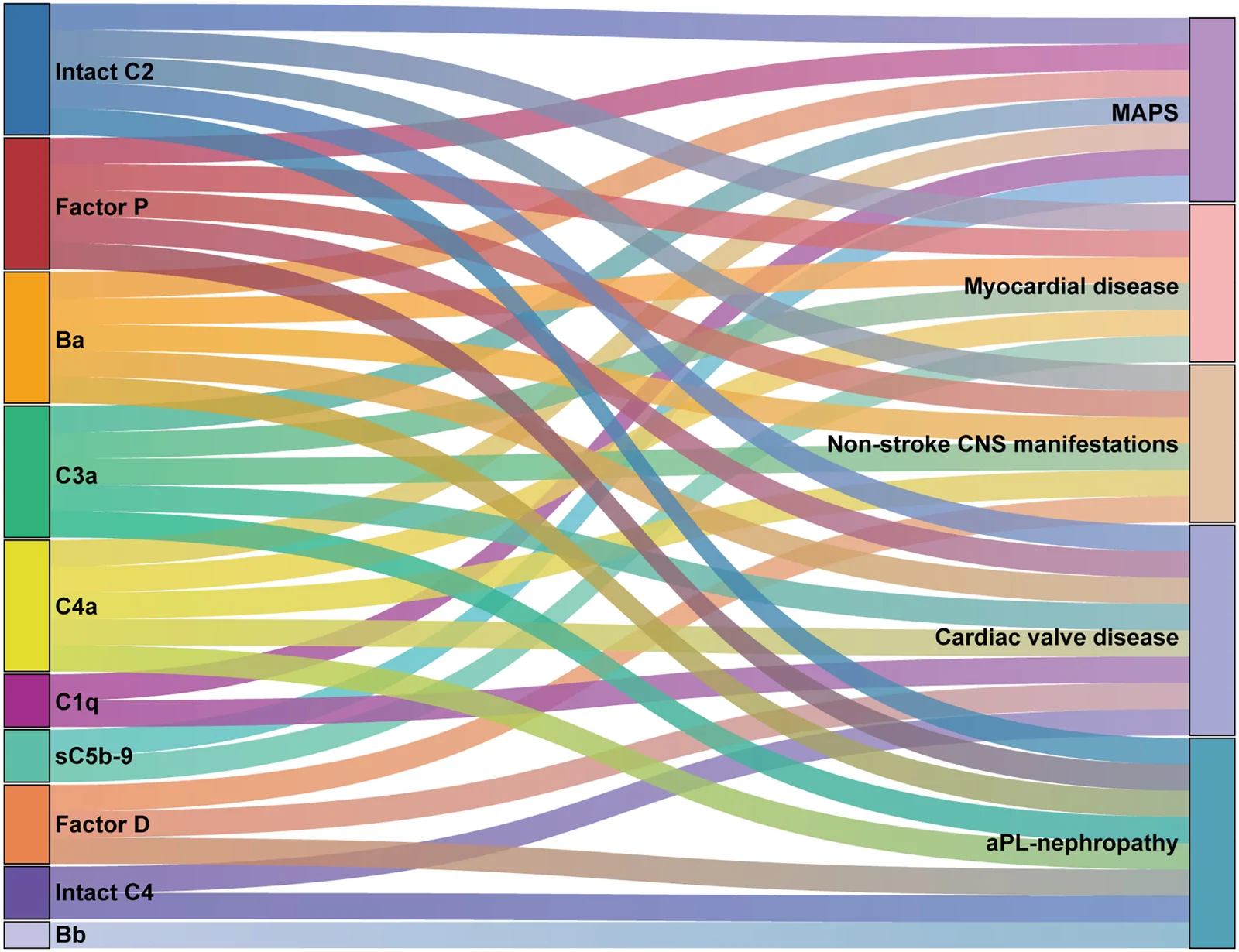
Associations between complement components and MAPS-related manifestations. aPL, antiphospholipid antibody; MAPS, microvascular antiphospholipid syndrome.

### 3.4 Clinical subgroups by cluster analysis

Cluster analyses revealed 3 subgroups with distinct phenotypic and serologic features (Figure 4). There was no significant difference in sex and age across subgroups (Supplementary Table 2).

**Figure 4:**
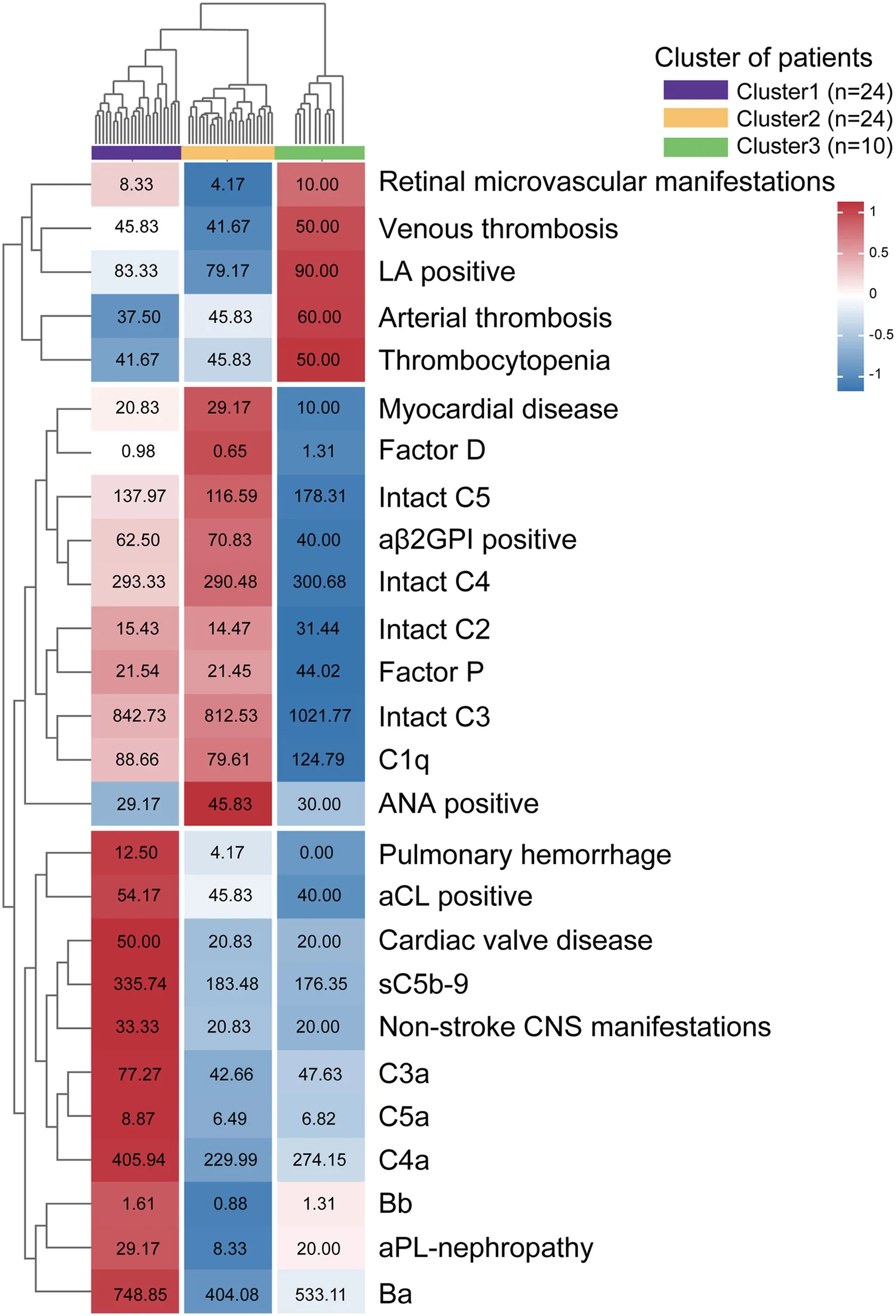
Complement levels and clinical characteristics of patients with APS in the 3 clusters. Cluster analysis identified 3 patient clusters (cluster 1, 2 and 3) and 3 variable clusters in patients with APS (n = 58). Complement levels are represented as mean or median, and clinical manifestations are represented as frequencies. aCL, anticardiolipin antibody; aPL, antiphospholipid antibody; APS, antiphospholipid syndrome; ANA, antinuclear antibody; CNS, central nervous system; HC, healthy control; LA, lupus anticoagulant.

Cluster 1 (n = 24, 41.38%) had the highest percentages of MAPS manifestations, including pulmonary hemorrhage, nonstroke CNS manifestations, aPL nephropathy, and cardiac valve disease. Intriguingly, cluster 1 showed the most significantly increased levels of Ba, Bb, C3a, C4a, and sC5b-9 among the 3 subgroups, indicating the strongest complement activation, with most prominent activation of the alternative pathway. Accordingly, glucocorticoids were most frequently used in cluster 1.

Cluster 2 (n = 24, 41.38%) was characterized by the highest frequency of myocardial disease, together with the highest positivity of ANA and aβ2GPI antibodies. Cluster 2 exhibited the lowest levels of C1q, intact C2, C3, C4, C5, and factor D, suggesting predominant activation of the classical and lectin pathways. Additionally, patients in this cluster most frequently received antiplatelet agents and immunosuppressants.

Cluster 3 (n = 10, 17.24%) was characterized by the highest prevalence of macrovascular thrombosis (arterial and venous) and thrombocytopenia, with the highest positivity of LA and relatively mild complement activation. The frequency of retinal microvascular manifestations was also mildly elevated in this cluster. Anticoagulants and hydroxychloroquine were more commonly used in these patients.

### 3.5 Establishment and evaluation of the predictive model for MAPS

Factor P and C3a were incorporated into the final predictive model for MAPS, and a nomogram was subsequently constructed (Figure 5A). The predictive formula derived from the nomogram was as follows: MAPS Score = −2.26805 − 0.00006×Factor P + 0.11303 × C3a. The performance of the nomogram was measured by ROC curve analysis. The area under the ROC curve (AUC) of this nomogram was 0.898, indicating good discriminative ability for MAPS from the combined group of patients with non-MAPS APS and healthy controls, with a sensitivity of 82.4% and a specificity of 88.9% (Figure 5B).

**Figure 5:**
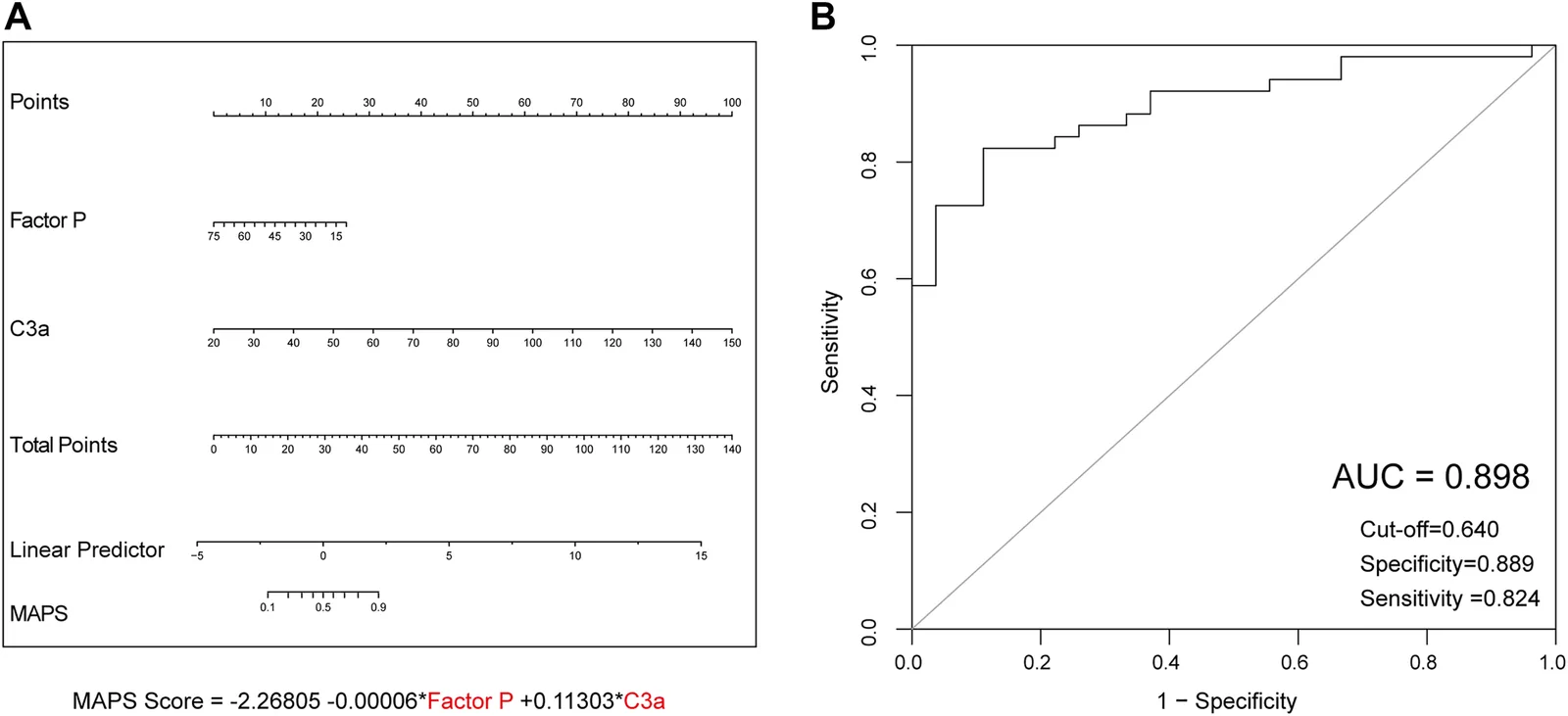
Establishment and evaluation of the predictive model for MAPS. (A) Nomogram for MAPS prediction. (B) Receiver operating characteristic curve of the predictive model. AUC, area under the receiver operating characteristic; MAPS, microvascular antiphospholipid syndrome.

The stepwise nomogram was further validated using internal bootstrap validation. The bootstrap-corrected AUC was 0.896 based on 1000 resamples and was comparable to that of the initial stepwise model (Supplementary Figure 1A). Calibration analysis, comparing predicted probabilities with observed outcomes, showed good agreement between the predicted and observed probabilities (Supplementary Figure 1B).

## 4 Discussion

To our knowledge, this is the first study to comprehensively characterize complement activation in patients with APS, focusing particularly on those with MAPS. Compared with HCs, patients with MAPS exhibited distinct patterns of complement activation, with decreased levels of C1q, C2, and factor P, together with increased Ba, C3a, C4a, and sC5b-9. Notably, compared with non-MAPS APS, patients with MAPS showed decreased C2 and factor P levels, as well as elevated C3a, Ba, and Bb levels. In addition, cluster analyses identified 3 phenotypically and serologically distinct APS subgroups, highlighting the heterogeneity of APS. Furthermore, by constructing a clinical prediction model, we found that factor P from the alternative pathway combined with C3a demonstrated good ability to discriminate MAPS. Collectively, these findings reveal the prevalent activation of all 3 complement pathways, particularly the alternative pathway, in the pathogenesis of MAPS, and underscore the predictive potential of complement biomarkers for patient stratification and precision management in APS.

Currently, there is a lack of reliable biomarkers to predict the presence of MAPS in patients with APS. Because MAPS is more insidious with worse clinical outcomes, and because it represents a phenotypically and mechanistically distinct entity from moderate-to-large vessel APS manifestations, the 2023 ACR/EULAR APS classification criteria formally recognized MAPS as an important clinical domain [6]. This update underscores the growing recognition of MAPS, a clinical subtype that had long been underappreciated. Complement activation has been well established as a critical driver of APS. In animal models, passive transfer of aPL IgG from patients with APS to mice primed with bacterial lipopolysaccharide could induce thrombosis, with C3 and C9 colocalized with aPL IgG on the mesenteric vessels. Blocking C5 or C6 markedly attenuated thrombosis [25], and C6-deficient mice were protected from thrombophilia [26]. Furthermore, complement activation has also been reported to be closely involved in the pathogenesis of MAPS, C5a and sC5b-9 have been found to predominate in patients with APS with small-vessel thrombosis [12], and patients with CAPS with renal involvement showed a high response (83.3%) to eculizumab [27], indicating the potential role of complement activation in MAPS, such as aPL nephropathy. However, previous studies have mainly focused on terminal complement components in APS (such as C5a and sC5b-9), which could not reflect the specific activation pathway. This study revealed for the first time that all 3 complement pathways are activated to varying degrees in MAPS, with particularly pronounced activation of the alternative pathway, which therefore broadens the understanding of complement involvement beyond the classic manifestations of APS.

Organ involvement in MAPS could be associated with distinct patterns of complement activation. Although previous studies have implicated terminal complement components in MAPS [27], the specific complement pathways contributing to different microvascular manifestations have not been systematically investigated. The complement cascade is composed of ∼50 circulating and membrane-associated proteins [28], which are highly labile and rapidly responsive to stimuli. Conventional diagnostic assays typically assess only a limited number of components, offering an incomplete picture of the cascade [29]. In addition, routine C3 and C4 testing has limited diagnostic and monitoring value in autoimmune diseases such as SLE [30]. For instance, in a prospective cohort of 71 lupus nephritis patients, C3 and C4 levels remained within normal ranges in one-third of patients, despite sensitivities for flare detection of 75% and 48%, respectively [31]. Similarly, no significant differences were observed in C3 and C4, commonly tested in routine clinical practice, across the clinical subgroups in this study. On the contrary, other complement components, such as C2, factor P, Ba, C3a, and C4a, exhibited more pronounced differences, indicating they may have greater clinical relevance than C3 and C4 in MAPS. Therefore, limited complement profiling may overlook key complement pathway-specific abnormalities relevant to organ manifestations of MAPS. Intriguingly, recent advance in multiplex assays require only microliter-scale plasma volumes and allow parallel measurement of multiple analytes, offering a comprehensive snapshot of complement activity in a single run. In the present study, we measured 15 complement components, enabling systematic profiling of MAPS and detailed evaluation of the association between complement activation pathways and organ-specific involvement. In the manifestation of myocardial lesions, the decrease in C2 and the increase of C4a suggest that both the classical and lectin pathway activation may mediate the myocardial lesions in MAPS. In nonstroke CNS manifestations, the elevation of Bb and factor D indicates that the overactivation of the alternative pathway is a key mechanism for CNS microvascular damage. In cardiac valve diseases, the reduction of C1q, C2, C4, and factor P, as well as the increase of factor D and Ba, suggested that both the classical and the alternative pathway converge to mediate valve lesions. These findings indicate that complement dysregulation is not uniform in different types of MAPS manifestations, reflecting distinct organ-specific pathogenic mechanisms.

Distinct patterns of complement activation were further delineated across APS subgroups identified by cluster analyses. Cluster 1, characterized by the highest burden of MAPS manifestations, and exhibited the strongest complement activation, with more pronounced activation of the alternative pathway than the classical and lectin pathways. Emerging evidence has shown that MAPS are often refractory to anticoagulants due to their predominantly immuno-inflammation–driven pathophysiology [32]. Case reports and series have revealed that traditional or novel immunosuppressive agents can be effective for certain MAPS manifestations [18]. Complement hyperactivation in cluster 1 likely induced severe systemic involvement and therefore glucocorticoids were most frequently used. Cluster 2 was enriched for myocardial disease and positivity for ANA and aβ2GPI antibodies, suggesting a higher likelihood of concomitant connective tissue diseases, such as SLE. The reduced C1q, C2, C3, C4, C5, and factor D levels in cluster 2 suggested predominant activation of the classical and lectin pathways rather than the alternative pathway. Given that β2GPI is the primary antigen for pathogenic aPLs [33], immune complexes containing aβ2GPI antibodies in cluster 2 may contribute to the ignition of the classical pathway. In contrast, cluster 3 was dominated by macrovascular thrombosis, thrombocytopenia, and high LA positivity but only mild complement activation, underscoring the divergent immunopathogenic mechanisms between thrombotic phenotypes and MAPS. Actually, enzymes of the contact system, such as kallikrein and factor XII (FXII), can trigger complement activation [34]. Activated FXII converts prekallikrein into kallikrein, which subsequently cleaves C3 to generate C3a and C3b, and also cleaves factor B to produce Ba and Bb [35]. Thus, the kallikrein-mediated activation pathway converges with the amplification loop of the alternative pathway. Conversely, complement components such as C1q, C3, and C5 [36], together with membrane-binding lectins (MBLs) and MBL-associated serine proteases (MASPs) [37], [38], contribute to thrombus formation. Furthermore, the terminal complement complex (C5b-9) enhances procoagulant activity by promoting platelet prothrombinase [39] and can also induce vesiculation of endothelial membranes, thereby exposing catalytic surfaces that facilitate prothrombinase complex assembly and fibrin deposition during immune-mediated endothelial injury [40]. These observations emphasize the critical interplay between complement activation and thrombogenesis. Importantly, complement activation tends to coincide with acute thrombotic episodes and is more likely to be detected near the timepoint of thrombosis [41]. Accordingly, the relatively mild complement activation in cluster 3 could be, at least partially, attributed to the quiescent phases of patients with APS with macrovascular thrombosis included in the current study. Although the 3 clusters were not clearly associated with major conventional APS manifestations, they showed distinct complement activation patterns, suggesting heterogeneity in complement involvement among patients with MAPS. Overall, the identified complement profiles may provide additional insight into the heterogeneity of MAPS. In particular, patients with marked complement activation may represent a subgroup of interest for future studies evaluating complement-targeted therapies. These findings should therefore be considered exploratory and require further validation in larger cohorts.

The 2023 ACR/EULAR classification criteria for MAPS require histopathologic confirmation in addition to imaging evidence, making the diagnosis of MAPS particularly challenging. Accordingly, prior studies have explored reliable predictive biomarkers for early identification of MAPS. Serum calprotectin positivity has been reported to be independently associated with MAPS (OR 1.90) and could predict MAPS in patients who were aPL positive (OR 1.83) [42]. Patients with elevated remnant cholesterol levels (>0.60 mmol/L) exhibited significantly increased risks for MAPS, with a multivariable-adjusted hazard ratio of 4.36 [43]. Furthermore, persistently high sC5b-9 and C5a levels during quiescent phases identified patients with APS at risk for more severe disease and recurrent thrombosis [12]. Chaturvedi et al. [41] reported that 60% of patients with CAPS carried rare germline variants in genes involved in complement regulation, such as complement factor H-related family, thrombomodulin, and complement receptor 1. In this study, we found that factor P levels were reduced in patients with MAPS compared with both patients with no-MAPS and HCs. This finding parallels reports in ANCA-associated vasculitis, where factor P levels decrease during active disease relative to remission [44]. Factor P is a well-established positive regulator of the alternative pathway, functioning by stabilizing C3 and C5 convertases and may also act as a pattern recognition molecule initiating alternative pathway activation [45]. Lower factor P levels in patients with MAPS may be related to activation and consumption of the alternative pathway. However, other factors, such as individual differences in complement synthesis or genetic background, may also influence circulating complement levels. Given the cross-sectional design of the current study, longitudinal changes in complement activation could not be evaluated. In addition, some patients in the cohort had concomitant autoimmune diseases, which may also have affected complement activation patterns. Although adjustment analyses were performed, the potential influence of treatment on complement activation could not be completely excluded. We further developed a predictive model incorporating factor P, a key regulator of the alternative pathway, together with C3a, which showed good discriminatory performance for MAPS in the current cohort. However, given the relatively small sample size and lack of external validation, these findings should be interpreted cautiously. Further studies are needed to determine the clinical utility of complement biomarkers in MAPS. Nevertheless, these findings support the potential value of complement biomarkers in identifying patients with APS at risk for MAPS and warrant further investigation in future studies.

There are several limitations in this study. Firstly, this study was conducted at a single center with a relatively small sample size, race, and ethnicity were not systematically recorded, which may limit the generalizability of our findings. In addition, the clustering findings should be interpreted with caution and require validation in larger cohorts. The predictive model was internally validated within the current cohort and has not yet been tested in independent external populations. Secondly, complement profiles were measured at a single baseline time point due to the cross-sectional design, without longitudinal dynamics, precluding causal inference. Reduced circulating complement levels could not be definitively distinguished from altered complement synthesis or baseline inter-individual variability. Thirdly, lectin pathway-specific components, such as MBLs and MASPs, were not measured in this study, resulting in an incomplete characterization of the complement profile. Finally, functional assays of complement and mechanistic investigations were lacking, and the precise role of complement activation in MAPS pathogenesis remains unclear. Future longitudinal studies are required to validate the predictive value of complement biomarkers for MAPS and to assess their utility in guiding therapeutic strategies.
