# Supplementary material for: Multiplex complement profiling uncovers complement activation signatures in microvascular manifestations of antiphospholipid syndrome
Source: Res Pract Thromb Haemost. 2026 Oct 1;10(7):106996. doi: 10.1016/j.rpth.2026.106996 (PMC13760085; doi:10.1016/j.rpth.2026.106996)
Supplement: Supplementary file 1 [file mmc1.docx]

**Supplementary Materials**

**Supplementary Table 1. Multivariable regression analyses of the associations between complement levels and MAPS**

| **Exposure** | **Model 1**  **OR (95%CI) P-value** | **Model 2**  **OR (95%CI) P-value** | **Model 3**  **OR (95%CI) P-value** |
| --- | --- | --- | --- |
| C1q | 0.98 (0.96, 1.00) 0.1089 | 0.98 (0.96, 1.00) 0.1076 | 0.99 (0.96, 1.02) 0.6192 |
| C2 | 1.00 (1.00, 1.00) 0.0030 | 1.00 (1.00, 1.00) 0.0026 | 1.00 (1.00, 1.00) 0.0173 |
| C3 | 1.00 (1.00, 1.00) 0.8942 | 1.00 (1.00, 1.00) 0.8723 | 1.00 (1.00, 1.01) 0.9023 |
| C4 | 0.95 (0.89, 1.02) 0.1375 | 0.95 (0.89, 1.02) 0.1722 | 0.95 (0.84, 1.08) 0.4501 |
| C5 | 1.00 (0.99, 1.02) 0.5798 | 1.00 (0.99, 1.02) 0.5729 | 1.00 (0.98, 1.02) 0.8856 |
| Factor D | 1.00 (1.00, 1.00) 0.1801 | 1.00 (1.00, 1.00) 0.1809 | 1.00 (1.00, 1.00) 0.5274 |
| Factor P | 1.00 (1.00, 1.00) 0.0016 | 1.00 (1.00, 1.00) 0.0015 | 1.00 (1.00, 1.00) 0.0428 |
| Ba | 1.01 (1.00, 1.01) 0.0010 | 1.01 (1.00, 1.01) 0.0012 | 1.01 (1.00, 1.01) 0.0320 |
| Bb | 3.16 (1.08, 9.27) 0.0364 | 3.22 (1.07, 9.67) 0.0367 | 4.60 (0.74, 28.47) 0.1007 |
| C3a | 1.12 (1.06, 1.19) <0.0001 | 1.13 (1.06, 1.20) <0.0001 | 1.09 (1.03, 1.16) 0.0041 |
| C4a | 1.00 (1.00, 1.01) 0.0363 | 1.00 (1.00, 1.01) 0.0407 | 1.00 (1.00, 1.01) 0.3215 |
| Factor I | 1.00 (1.00, 1.00) 0.2512 | 1.00 (1.00, 1.00) 0.2045 | 1.00 (1.00, 1.00) 0.9778 |
| Factor H | 1.00 (0.99, 1.00) 0.5432 | 1.00 (0.99, 1.00) 0.6443 | 0.99 (0.98, 1.00) 0.1823 |
| C5a | 0.99 (0.86, 1.13) 0.8529 | 0.99 (0.86, 1.14) 0.9077 | 0.96 (0.76, 1.20) 0.6932 |
| sC5b-9 | 1.01 (1.00, 1.02) 0.0214 | 1.01 (1.00, 1.02) 0.0218 | 1.00 (0.99, 1.01) 0.3453 |

Model 1: No covariates were adjusted.

Model 2: Age and sex were adjusted.

Model 3: Age, sex, anticoagulant therapy, antiplatelet therapy, glucocorticoid use, immunosuppressive treatment, and hydroxychloroquine exposure were adjusted.

MAPS: microvascular antiphospholipid syndrome; OR: odds ratio

**Supplementary Table 2. Different complements levels and clinical characteristics of APS patients in the three clusters**

|  | **Cluster 1** | **Cluster 2** | **Cluster 3** | ***P*-value** |
| --- | --- | --- | --- | --- |
| **N (%)** | 24 | 24 | 10 |  |
| Female, n (%) | 16 (66.67) | 16 (66.67) | 5 (50.00) | 0.61 |
| Age (years) (median (IQR)) | 38.00 (30.00-46.75) | 34.00 (29.75-40.50) | 40.00 (35.00-44.00) | 0.19 |
| ANA positive, n (%) | 7 (29.17) | 11 (45.83) | 3 (30.00) | 0.44 |
| Venous thrombosis, n (%) | 11 (45.83) | 10 (41.67) | 5 (50.00) | 0.90 |
| Arterial thrombosis, n (%) | 9 (37.50) | 11 (45.83) | 6 (60.00) | 0.48 |
| Pregnancy morbidity^§^, n (%) | 1 (6.25) | 3 (18.75) | 0 (0.00) | 0.33 |
| Cardiac valve disease, n (%) | 12 (50.00) | 5 (20.83) | 2 (20.00) | 0.06 |
| Thrombocytopenia, n (%) | 10 (41.67) | 11 (45.83) | 6 (60.00) | 0.62 |
| **Microvascular disease, n (%)** |  |  |  |  |
| Livedo racemose | 9 (41.67) | 9 (50.00) | 0 (00.00) | <0.01 |
| aPL-nephropathy | 7 (29.17) | 2 (8.33) | 2 (20.00) | 0.18 |
| Pulmonary hemorrhage | 3 (12.50) | 1 (4.17) | 0 (0.00) | 0.33 |
| Myocardial disease | 5 (20.83) | 7 (29.17) | 1 (10.00) | 0.46 |
| Retinal microvascular manifestations | 2 (8.33) | 1 (4.17) | 1 (10.00) | 0.78 |
| Non-stroke CNS manifestations | 8 (33.33) | 5 (20.83) | 2 (20.00) | 0.55 |
| **APL profiles, n (%)** |  |  |  |  |
| Positive LA (persistent) | 20 (83.33) | 19 (79.17) | 9 (90.00) | 0.75 |
| aCL IgM medium/high titer | 1 (4.17) | 1 (4.17) | 2 (20.00) | 0.20 |
| aCL IgG medium/high titer | 12 (50.00) | 10 (41.67) | 2 (20.00) | 0.27 |
| aβ2GPI IgM medium/high titer | 4 (16.67) | 5 (20.83) | 2 (20.00) | 0.38 |
| aβ2GPI IgG medium/high titer | 13 (54.17) | 13 (54.17) | 3 (30.00) | 0.93 |
| **Treatments, n (%)** |  |  |  |  |
| Antiplatelet | 10 (41.67) | 12 (50.00) | 2 (20.00) | 0.27 |
| Anticoagulant | 16 (66.67) | 13 (54.17) | 8 (80.00) | 0.34 |
| Hydroxychloroquine | 17 (70.83) | 20 (83.33) | 9 (90.00) | 0.37 |
| Glucocorticoid | 19 (79.17) | 16 (66.67) | 4 (40.00) | 0.09 |
| Immunosuppressant | 12 (50.00) | 14 (58.33) | 4 (40.00) | 0.61 |
| **Complement test** |  |  |  |  |
| C1q (μg/mL) (mean ± SD) | 88.66 ± 13.25 | 79.61 ± 13.05 | 124.79 ± 26.54 | <0.01 |
| Intact C2 (μg/mL) (mean ± SD) | 15.43 ± 5.36 | 14.47 ± 3.23 | 31.44 ± 7.27 | <0.01 |
| Intact C3 (μg/mL) (median (IQR)) | 842.73 (813.25-904.84) | 812.53 (785.31-849.34) | 1021.77 (967.11-1195.45) | <0.01 |
| Intact C4 (μg/mL) (median (IQR)) | 293.33 (291.62-294.85) | 290.48 (289.21-292.13) | 300.68 (296.51-303.74) | <0.01 |
| Intact C5 (μg/mL) (median (IQR)) | 137.97 (126.74-150.00) | 116.59 (103.30-125.24) | 178.31 (151.13-194.10) | <0.01 |
| Factor D (μg/mL) (median (IQR)) | 0.98 (0.79-1.30) | 0.65 (0.52-0.75) | 1.31 (1.08-1.61) | <0.01 |
| Factor P (μg/mL) (median (IQR)) | 20.92 (16.58-26.17) | 21.38 (17.98-24.60) | 41.34 (38.04-48.59) | <0.01 |
| Ba (ng/mL) (median (IQR)) | 748.85 (645.53-897.16) | 404.08 (342.95-515.89) | 533.11 (488.90-621.39) | <0.01 |
| Bb (μg/mL) (median (IQR)) | 1.61 (1.44-1.95) | 0.88 (0.72-1.08) | 1.31 (1.01-1.50) | <0.01 |
| C3a (ng/mL) (median (IQR)) | 77.27 (49.58-90.74) | 42.66 (36.20-56.94) | 47.63 (33.11-54.25) | <0.01 |
| C4a (ng/mL) (median (IQR)) | 405.94 (297.62-613.78) | 229.99 (167.96-296.50) | 274.15 (193.78-552.48) | <0.01 |
| Factor I (μg/mL) (median (IQR)) | 26.63 (25.18-29.59) | 20.010 (16.54-24.98) | 27.69 (22.38-33.36) | <0.01 |
| Factor H (μg/mL) (median (IQR)) | 277.50 (255.10-319.32) | 279.67 (233.95-321.56) | 284.92 (265.31-356.71) | 0.55 |
| C5a (ng/mL) (mean ± SD) | 8.87 ± 4.25 | 6.49 ± 3.03 | 6.82 ± 2.28 | 0.15 |
| sC5b‐9 (ng/mL) (median (IQR)) | 335.74 (230.80-471.08) | 183.48 (176.00-265.15) | 176.35 (176.00-208.09) | <0.01 |

^§^Meeting the pregnancy morbidity criteria of the 2006 revised Sydney APS classification.

APS: antiphospholipid syndrome; MAPS: microvascular antiphospholipid syndrome; HC: healthy control; ANA: antinuclear antibody; aPL: antiphospholipid antibody; aCL: anticardiolipin antibody; aβ2GPI: anti-β2-glycoprotein I; LA: lupus anticoagulant; CNS: central nervous system; IQR: interquartile range; N/A: not available or not applicable.

**Supplementary Figure 1. Internal validation of the nomogram using bootstrap sampling.**

(A) Receiver operating characteristic curve was assessed by bootstrapping for 1000 repetitions, demonstrating the AUC of the stepwise model; (B) Calibration curve comparing predicted probabilities with observed outcomes for the MAPS nomogram.

AUC: area under the receiver operating characteristic curve; ROC: Receiver operating characteristic; MAPS: microvascular antiphospholipid syndrome
